# The past, present and future of ancient bacterial DNA

**DOI:** 10.1099/mgen.0.000384

**Published:** 2020-06-29

**Authors:** Nicolas Arning, Daniel J. Wilson

**Affiliations:** ^1^​ Big Data Institute, Nuffield Department of Population Health, University of Oxford, Li Ka Shing Centre for Health Information and Discovery, Old Road Campus, Oxford, OX3 7LF, UK

**Keywords:** ancient bacterial DNA, ancient pathogens, bacterial evolution, paleogenetics, paleomicrobiology

## Abstract

Groundbreaking studies conducted in the mid-1980s demonstrated the possibility of sequencing ancient DNA (aDNA), which has allowed us to answer fundamental questions about the human past. Microbiologists were thus given a powerful tool to glimpse directly into inscrutable bacterial history, hitherto inaccessible due to a poor fossil record. Initially plagued by concerns regarding contamination, the field has grown alongside technical progress, with the advent of high-throughput sequencing being a breakthrough in sequence output and authentication. Albeit burdened with challenges unique to the analysis of bacteria, a growing number of viable sources for aDNA has opened multiple avenues of microbial research. Ancient pathogens have been extracted from bones, dental pulp, mummies and historical medical specimens and have answered focal historical questions such as identifying the aetiological agent of the black death as *Yersinia pestis *. Furthermore, ancient human microbiomes from fossilized faeces, mummies and dental plaque have shown shifts in human commensals through the Neolithic demographic transition and industrial revolution, whereas environmental isolates stemming from permafrost samples have revealed signs of ancient antimicrobial resistance. Culminating in an ever-growing repertoire of ancient genomes, the quickly expanding body of bacterial aDNA studies has also enabled comparisons of ancient genomes to their extant counterparts, illuminating the evolutionary history of bacteria. In this review we summarize the present avenues of research and contextualize them in the past of the field whilst also pointing towards questions still to be answered.

## Introduction

Despite the growing wealth of genomic data since the advent of next-generation sequencing, some domains of life remain elusive for evolutionary biologists [1]; in particular, the past of bacteria, the oldest domain [2], is inaccessible due to a poor fossil record [3]. In lieu of fossil evidence, the study of bacterial evolution has benefited greatly from the use of ancient DNA (aDNA) extracted from archaeological specimens. Paleogenetics, the study of aDNA, has grown steadily alongside technical advancements in genetics, from the amplification of single loci in the 1980s to yielding 34 complete ancient genomes in a recent study [4]. aDNA has opened an increasingly larger window into the past, answered fundamental questions concerning human evolution [5] and uncovered otherwise inscrutable bacterial history. Here, we chronologically review how paleogenetics has helped the understanding of bacterial evolution covering human pathogens and microbiomes as well as environmental microbes. As an introduction we contextualize the present state of research relative to the field’s past and highlight major breakthroughs and setbacks in its trajectory. We subsequently encapsulate the present state of the field by providing a snapshot of current findings and a catalogue of available paleogenomes. Lastly, we assess the future of bacterial aDNA through possible avenues of investigation that provide potential starting points for future endeavours using bacterial aDNA.

## THE PAST: GROWTH AND SETBACKS OF THE FIELD

### The birth of a field

The first breakthrough in aDNA occurred when Higuchi *et al.* [6] were able to extract a small genetic fragment from a 150-year-old museum specimen of the extinct horse relative quagga through laborious bacterial cloning. Paleogenetics was soon propelled by the development of the polymerase chain reaction (PCR) [7]. PCR produces abundant genetic material from infinitesimal amounts of aDNA given a primer to guide the amplification. With the choice of primer, researchers can decide whether they want to take a targeted approach to generating aDNA from ideally a single species (e.g. amplifying a gene of a specific toxin), or an untargeted approach generating aDNA from multiple species (e.g. amplifying 16S rDNA found in all bacteria). The success of amplification on human, animal and plant remains spurred the first bacterial aDNA studies targeting *Mycobacterium tuberculosis* [8] and *Mycobacterium leprae* [9]. Müller and colleagues [10], however, highlight that the presence of mycobacteria from the human microbiome or soil could lead to false positives without further scrutiny, casting doubt on the validity of early findings lacking subsequent authentication. Not only did the extraction of mycobacteria prove difficult, but the whole field quickly reached limits imposed by the then available technology because PCR is far from optimal for analysing aDNA. Successful amplification requires relatively long, well-preserved, sequences that cannot easily be acquired from very short degraded ancient samples [11] (typically shorter than 100 base-pairs [12]). Additionally, primers for aDNA amplification must be specifically tailored to ancient targets, which poses unique challenges in design. Using contemporary sequences, a section of DNA must be identified that is taxon-specific and short enough to be covered by short aDNA fragments, but also long enough to be used for amplification. Success is not guaranteed, given that mutations can occur that obstruct the design of viable primers for ancient material from modern-day counterparts. The amplification of minuscule ancient genetic material required for detection, coupled with difficulties of authentication, led to widespread contamination from modern-day DNA [13]. Due to the large number of PCR cycles needed for ancient samples, the procedure is liable to amplify trace amounts of modern-day and environmental DNA or that of postmortem colonizers [14]. Additionally, degradation of DNA over time can lead to sequencing errors because enzymes used for amplification preferentially bind to non-damaged modern contaminants [15]. The debate concerning the authenticity of early aDNA studies led to the development of rigorous validation processes that are still used today [12].

### The advent of high-throughput sequencing

The advent of high-throughput sequencing (HTS) rejuvenated the field of paleomicrobiology as sample and data output increased by orders of magnitude [12] and brought new possibilities for authentication. The mass of sequence data led to a growth spurt in the field of computational sequence analysis, allowing *in silico* verification based on sequence patterns [16]. Signatures of postmortem damage (PMD) create nucleotide misincorporations characteristic of aDNA [17]. Generally, two forms of characteristic mechanisms of PMD are used for verification: depurination and cytosine deamination [18]. In depurination purine bases are removed through the cleavage of a N-glycosyl bond, leaving an abasic site that is susceptible to subsequent β-elimination, which breaks the DNA backbone [19]. This fragmentation causes an overrepresentation of purine bases preceding strand breaks, which is a pattern unique to ancient DNA. Fragmentation also results in sequences that are typically shorter than 100 bp [20], which is additionally used to differentiate ancient sequences from modern contaminants harbouring longer sequences [12]. In cytosine deamination, cytosine loses an amine group and is thus converted to uracil, which is registered as thymine in sequencing [20]. Bases become predominantly deaminated towards the exposed single-stranded ends of fragments, leading to an increase of misincorporations towards the 5ʹ end, another distinctive signature used for authenticating aDNA [21]. Age is not the only contributing element in PMD, because factors such as temperature, depositional conditions, post-excavation handling and source tissue can also influence the signatures, so that contaminants may also exhibit damage patterns [21]. However, it was shown that younger (50–100 years old) samples exhibited little PMD and a linear correlation of damage with time was discovered [22]. Misincorporations and fragmentation can be detected through alignment of reads to extant bacterial genomes, where the removal of PCR duplicates and high coverage ensure that misincorporations can be distinguished from genuine substitutions. Provided there is a genome available for the organism of interest, alignment allows the depletion of modern contaminants lacking PMD signatures [23].

The development of targeted capture sequencing enabled enrichment using modern DNA or RNA as bait, making the recovery of ancient genetic material more financially viable [24–26]. Enrichment facilitates the sequencing of miniscule amounts of ancient material from a species of interest amidst within-sample (endogenous) contaminating sequences from other species and environmental (exogenous) modern sequences. Targeted enrichment led to the first ancient bacterial genome of *Yersinia pestis *, which was recovered from the teeth of a 14th-century plague victim at 30-fold coverage [27]. The isolation of a high-coverage *Y. pestis* paleogenome verified earlier work by Drancourt *et al.* [28]. This confirmation put to rest the controversy around the possibility of isolation of the plague bacterium from teeth [29], which was sparked by the negative findings of Gilbert *et al.* [20] and opened up the possibility of a comparative genomic approach using aDNA.

Comparisons using paleogenomes facilitate a better understanding of evolutionary processes through time, enabling the inference of ancient demography and admixture, within-population evolution and calibration of the molecular clock [12]. In clock calibration the evolutionary rate is quantified, which describes genetic change as a function of time and in turn enables the dating of evolutionary events [30]. For accurate rate estimation, chronological anchors need to be supplied, which is especially fraught with difficulty for bacteria, since fossil evidence is nonexistent [31]. Further uncertainty is contributed by confounding factors such as the dependence of rate estimates upon the time spanned by sampling. Rates estimated by comparing species that have diverged millions of years ago are orders of magnitude slower than rates estimated within lineages that have diverged over decades [32]. This temporal inconsistency was named the time-dependent rate phenomenon and leads to discord concerning the timing of evolutionary events, because the interpolation of rates across different time periods is contentious [33]. Reported bacterial rate estimates reveal a broad spectrum between 10 substitutions per megabase per year (Mby^−1^) in *Neisseria gonorrhoeae* [34] and 0.001 Mby^−1^ in *M. tuberculosis* [35]. Chronologically, aDNA provides sequences dating within the gap that spans rate estimates drawn from comparison between and within species [33]. Therefore, datasets that comprise contemporary and ancient samples are apt to address the conflict in reported evolutionary rates. Bos and colleagues [36] investigated the impact of the inclusion of aDNA of increasingly larger time periods using three different *Y. pestis* datasets. The estimated mean ages were pushed further back the older the included sequences were and showed that the inclusion of Late Neolithic and Bronze Age samples decreased dating uncertainty immensely. Duchêne *et al.* [37] analysed aDNA from three different species (* M. leprae* from Schuenemann *et al.* [38], *M. tuberculosis* from Bos *et al.* [39] and *Y. pestis* from Wagner *et al.* [40]) spanning approximately 2000 years and confirmed the existence of a rate phenomenon. Where applicable, the discord between rate estimates spanning different time periods will be detailed in the following, as it provides an important application of aDNA in bacterial genomics, a field particularly impacted on by incongruity.

**Fig. 1.**
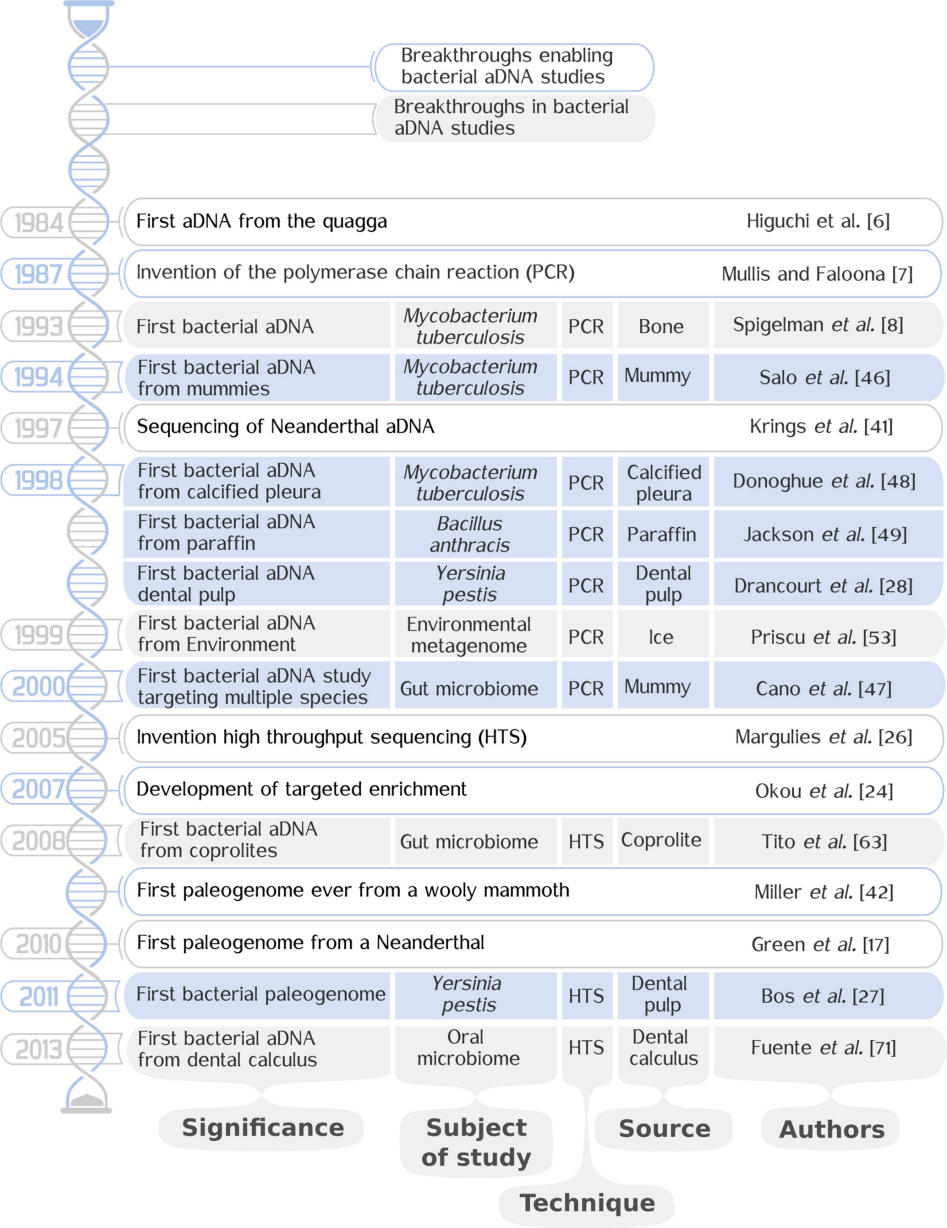
The history of bacterial aDNA research. Listed chronologically here are notable events in the history of bacterial aDNA research. Additionally, breakthroughs that are not bacterial aDNA studies, but have enabled research within the field, are listed to provide context. Breakthroughs in bacterial studies are highlighted in shaded blue and grey rows, with the significance, subject of study, technique of retrieval, source of genetic material and authors listed. Breakthroughs in paleogenetics not focusing on bacteria are in rows with white background.

Alongside high-profile extraction like Neandertal [41] and woolly mammoth [42] DNA, bacterial aDNA research grew steadiliy (Fig. 1) from the study of 16S rDNA of a single species to the recovery and comparison of full genomes from multiple genera. The history of bacterial aDNA research (Fig. 1) has led from the study of 16S rDNA of a single species to the recovery and comparison of full genomes from multiple genera. The key methodological stepping stones for paleogenetics are the development of PCR and HTS, leading to key publications such as the first amplification of bacterial aDNA [8] and the first retrieval of a full bacterial paleogenome [27]. Duchêne and colleagues’ study [37] is also an important achievement, where aDNA was first used across a range of species to make more comprehensive statements about the history of the bacterial domain. Also vital for analysing the bacterial past is the discovery of a growing collection of viable sources for the retrieval of aDNA, each with its distinct opportunities and obstacles.

### Sources of bacterial aDNA

The beginning of sequencing, and therefore also paleogenetics, was facilitated by targeted PCR amplification, making the identification of candidate targets from ancient samples vital to the success of early attempts at acquiring bacterial aDNA. Seminal research was directed at the recovery of genetic material from bone as aDNA extraction is destructive and often unsuccessful. Bones are the most common of the invaluable and finite archaeological resources suited for aDNA retrieval. The choice of study organism fell upon pathogens causing diseases that can occasionally be identified by skeletal lesions, to further increase chances of successful extraction. The choice fell upon tuberculosis (TB) and leprosy, leading to the first retrieval of bacterial aDNA of *M. tuberculosis* by Spigelman *et al.* [8], followed by Rafi *et al.* [9] amplifying *M. leprae *. The realization that aDNA can be recovered from bone tissue without visual evidence of disease led to an increase in available sequences, facilitating epidemiological investigations of populations [43] and mixed infections [44].

The extraction of aDNA from *M. tuberculosis* soon moved from bones to mummies, where natural mummification can lead to preservation, leaving bacterial DNA sufficiently intact for amplification [45]. Salo *et al.* [46] were able to acquire bacterial DNA from a 1000–2000 year-old mummified Peruvian body. The study revealed pre-Columbian presence of TB in the Americas, a disease formerly believed to be introduced by explorers [39]. Another successful extraction of bacterial aDNA from mummies was conducted by Cano *et al.* [47] through amplification of 16S ribosomal DNA, genes common to all bacteria, providing the first glimpse into ancient microbiomes. The subject was the 5300-year-old exceptionally well-preserved Tyrolean iceman. Similarities to the modern human gut flora were found with members of the genus *Clostridium *. The concern around PCR amplification producing false positives makes the validity of these early findings questionable, especially because Cano and colleagues also found clostridia in the grass sample taken from the surroundings of the iceman.

Donoghue *et al.* [48] were able to add calcified pleura to the sources from which *M. tuberculosis* could be amplified with PCR. Furthermore, historic medical specimens, such as organs embedded in paraffin, were proven to be viable subjects for paleomicrobiology. Jackson *et al.* [49] amplified *Bacillus anthracis* DNA from anthrax victims from 1979. Devault *et al.* [50] later used the alcohol-preserved colon of an 1849 cholera victim to generate the first and only paleogenome of *Vibrio cholerae *.

The inventory of aDNA sources was broadened by Drancourt *et al.* [28], who realized that teeth are both easily retrievable from burial sites and highly vascularized and thus a resource for bloodborne diseases [51]. Teeth as a preserving molecular niche minimize the potential for contamination through postmortem colonization of bacteria [52]. Drancourt *et al.* were able to retrieve DNA from *Y. pestis *, and thus found the aetiological agent for the black death. They suggest that dental pulp, as a better protected niche than bones that is equally expendable, should be used to detect ancient pathogens.

Early claims of isolation of bacterial aDNA from the environment were made by Priscu *et al.* [53] and Christner *et al.* [54] through the amplification of 16S rDNA from meltwater and ice from Lake Vostok, Antarctica. Willerslev *et al.* [55], however, pointed out that one of the organisms identified is a common laboratory contaminant and the only species recovered in both studies was also found in PCR controls. Further doubt was cast by subsequent studies that were unable to recover bacterial DNA from Lake Vostok ice [56]. Willerslev *et al.* [57] addressed their own criticism and amplified 16S rDNA under stringent laboratory conditions. Their samples were frozen under optimal conditions in Antarctica and northern Siberia.

Besides mummified remains, ancient human microbiomes commonly endure only in two substrates: coprolites and dental calculus [58]. Coprolites are mummified or petrified faeces that can contain the composition of intestinal microbiota at the point of death, allowing the study of the gut microbiome [59]. As myriads of micro-organisms are active in faeces, decomposition is rapid, leading to quick sample degradation [58]. Although found in dry, cold and tropical environments [60, 61]**,** preservation is only optimal under extremely dry or frozen conditions [47]. The detection of coprolites is difficult due to partial fossilization or fragmentary or amorphous preservation [62]. Despite their rarity, Tito *et al.* [63] were able to use HTS on coprolites from 1300-year-old Mexican cave samples. Their findings suggest that human microbiomes were previously more geographically structured. Apart from bacteria, the parasitological composition can be assessed through the presence of eggs, larval protein and parasite DNA [58], while diet can be assessed through plant, fungal and faunal remains [64]. A combined analysis of diet, microbiota and parasites allows for estimates of ancient dietary habits, lifestyles and cultures [65]. The comparison of microbiomes via coprolites has revealed a similarity between ancient and extant rural samples that indicates a compositional shift of microbiota in the modern cosmopolitan lifestyle [66].

The third possible source for human microbiomes is dental calculus. Calculus consists of dental plaque, saliva and crevicular fluid forming calcified bacterial biofilms incrementally through deposition and mineralization [58, 67]. Composed of layered structures, calculus produces a serial record of the oral microbiome showing the individual’s life history of diet and disease [67, 68]. DNA preserves well in calculus when compared to other sources, with preliminary microscopy analyses revealing whole bacteria conserved as rods and cocci [69]. Preus *et al.* [70] first proved the existence of DNA in calculus through transmission electron microscopy, which led Fuente *et al.* [71] to use species-specific PCR primers on 16S rDNA from five bacterial species. Adler *et al.* [72] were the earliest to assess the full microbiome by using 16S rDNA primers. Comparison to modern samples showed a shift to a disease-associated composition of oral microbiomes during the Industrial Revolution, with dental caries and periodontal disease-linked bacteria *Streptococcus mutans* and *Porphyromonas gingivalis *. Warinner *et al.* [67] used HTS to reveal key virulence and antibiotic resistance genes present in a medieval German specimen. Quantifying DNA preparation showed that the extraction of 1000 times more aDNA was possible from calculus compared to bone from the same individual. The abundance of extractable aDNA combined with the dominance of bacteria within the sample makes calculus a rich resource for paleomicrobiology [67]. However useful, using calculus as a source also has limitations. Calculus limits species of interest to members of the human oral microbiome and possibly provides a skewed representation of the past microbial diversity. Through the comparison of modern dental plaque and modern dental calculus, Velsko *et al.* [73] showed that a systematic bias in microbial composition is introduced in the calcification process, calling the inferences of Adler *et al.* into question.

**Fig. 2.**
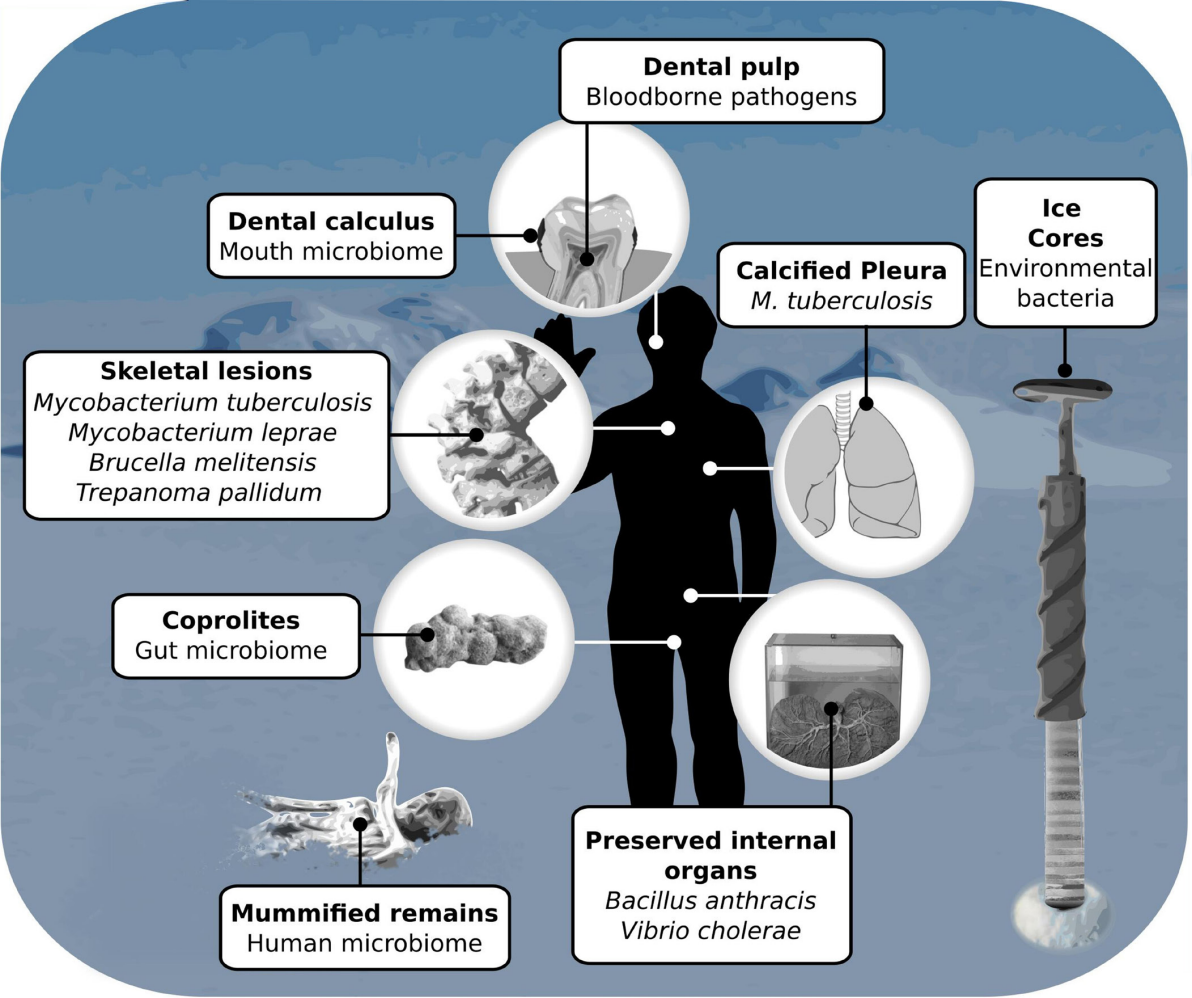
Sources of bacterial ancient DNA. The figure shows all currently viable sources for acquiring bacterial aDNA in bold. Below the sources the subjects of study that have been explored using these sources are listed.

Looking at all viable source of bacterial aDNA (Fig. 2), the initial study of Drancourt and colleagues should be highlighted as having discovered a valuable resource for human bloodborne pathogens. Dental pulp is better protected from contamination than bone, more easily available than coprolites and not as skewed in composition as calculus. Seminal studies in ice cores [53–57] should also be highlighted as the only source making ancient environmental bacteria accessible. Ice cores additionally have superior temporal reach compared to other sources [74], which is potentially crucial for resolving the discord in bacterial rate estimates and giving a glimpse of evolutionary dynamics outside a constrained host niche. Besides considerations of the source material for bacterial aDNA studies, the field faces systematic challenges unique to microbial research within archaeogenetics.

### Challenges in bacterial aDNA studies

Archaeological samples used in bacterial aDNA studies show a mixture of endogenous (ante-mortem) viable ancient material and exogenous (postmortem) sequences from colonization after death of the organism [12]. Endogenous DNA is made up of host-associated commensal taxa and epidemic pathogens [12]. Exogenous DNA contains environmental bacteria, contamination from human interaction with samples, suboptimal storage and laboratory contaminants [12]. Bacterial aDNA retrieval is prone to false positives, given that many pathogenic bacteria are members of the same genera as environmental bacteria [75]. Authentication via PMD can be thwarted by postmortem colonization at different ages, resulting in a range of damage patterns from ancient to extant that impedes distinction [76]. A key methodological advancement for overcoming the challenge of contamination was the development of mapDamage [77], which is a computational tool that analyses PMD signatures and allows the identification of aDNA within a chronologically heterogeneous genetic sample.

Other than false positives, assessing bacterial communities can be inherently problematic in paleomicrobiology. The equilibrium of the microbiome can shift quickly after death, with anaerobic bacteria persisting more successfully, which can lead to false conclusions about ancient microbiomes [78]. Bacterial aDNA studies are further altered by the differential conservation of bacterial DNA. GC content and the composition of the cellular membrane influences preservation, so that GC-rich Gram-positive bacteria persist best [65, 78]. Membranes rich in mycolic acid allow for better protection against enzymatic degradation, as is the case in mycobacteria [79]. Ziesemer *et al.* [80] highlighted that no study has systematically investigated how age-related degradation might shift the reconstruction of ancient microbial communities. Heeding their own advice, Ziesemer *et al.* looked at systematic amplification bias of the V3 regions in 16S rDNA. They showed more successful amplification for shorter V3 regions, which might have skewed earlier, untargeted aDNA studies.

Despite the inherent challenges they pose, some characteristics of bacteria favour the study of paleogenomes. The bacterial bauplan harbours unique benefits for preservation, such as increased resilience based on membrane composition. Glycosidic ether lipids and hopanoids are components of bacterial membranes not found in archaea or eukaryotes, which protect DNA from enzymatic degradation [81]. Intracellular DNA protected in dormant or dead microbes conserves better than naked DNA [65]. Aided by benefits in preservation, and despite challenges unique to bacteria, researchers have been able to generate a tremendous amount of insight from the study of bacterial aDNA.

## The present: insights gained from aDNA studies in bacteria

### Human pathogens

The development of agriculture brought food security and a settled lifestyle to human societies, but also led to an increase in infectious disease and is considered to be the origin of many important infections (see e.g. Harper *et al.* [82]). The progression to denser, more fertil,e human populations in proximity to domesticated animals was coined the Neolithic demographic transition [83]. Molecular dating has hitherto linked the emergence of at least three pathogens pivotal for paleogenetics with the Neolithic transition: *Y. pestis, M. tuberculosis* and *M. leprae* [84].

#### *Yersinia pestis*

As the causal agent of plague, *Y. pestis* is responsible for multiple human pandemics with millions of deaths through human history and is therefore a focal species for epidemiological research [85]. Plague can present itself in three forms: pneumonic, septicaemic and bubonic, where the latter is believed to have caused three historic plague pandemics: the first pandemic (also called the Justinian plague; 6th–8th century), the second pandemic (also called the medieval plague; 14th–18th century) and the third pandemic (also called modern plague; 19th century–present) [86].

The earliest infections with *Y. pestis* have been dated to 5000 years before present (YBP) by Rasmussen *et al.* [85] through the retrieval of paleogenomes from European and Asian Bronze Age teeth using HTS. The ancient strains from what was termed the Stone Age plague lacked key virulence factors of modern-day *Y. pestis *, as the *Yersinia* murine toxin gene *ymt* is absent in all samples older than 3700 years. Valtuena *et al.* [87] reconstructed six paleogenomes from 4800 to 3700 YBP, finding that the emergence of *Y *. *pestis* coincided with the ‘steppe ancestry’, where human expansion from central Eurasia to Eastern and Central Europe carried plague into Europe. The steppe ancestry of plague was confirmed by Damgaard *et al.* [88], who analysed 137 human paleogenomes along migratory routes, finding detectable levels of *Y. pestis* aDNA. Temporally, the emergence of *ymt* as suggested by Spyrou *et al.* [89] predates the arrival of bubonic plague in Europe, as determined by Valtuena *et al.* [87], which indicates that the acquisition of *ymt* could have caused a wide geographical spread originating in the steppe of Eurasia.

The first pandemic is the first recorded bubonic outbreak of *Y. pestis *, starting in the sixth century and lasting until the mid-eighth century [86]. The responsible strain was characterized by Wagner and colleagues [40], who recovered paleogenomes with targeted capture sequencing and HTS. The subjects came from burial sites in Germany, which were radiocarbon dated to within the first pandemic. The construction of a phylogenetic tree containing modern strains and paleogenomes from other historic pandemics showed the Justinian plague strain forming a separate branch, joining the rest of the tree prior to contemporary strains from China. This suggests that an extinct or under-sampled strain, possibly originating in China, was responsible for the Justinian plague but not for the subsequent pandemics. Multiple emergences of the plague from a rodent reservoir throughout history appear likely [40]. Keller *et al.* [90] reconstructed a further eight paleogenomes from the first pandemic. Their findings demonstrate a wide geographical range through Britain, France, Germany and Spain, but Keller and colleagues caution that the geographical origin of the first pandemic in Europe remains unresolved.

The second pandemic lasted from the 14th until the 18th century, with a peak mortality during the period of the Black Death from 1348 to 1351, killing an estimated 30–50 % of the European population [27]. Bos *et al.* [27] retrieved a *Y. pestis* paleogenome lying ancestral to extant strains from four 700-year-old English plague victims. Its position in the phylogenetic tree suggests that the second pandemic is the first appearance of contemporary *Y. pestis.* The medieval paleogenome revealed no unique derived positions when compared to modern strains. A lack of genetic divergence between Black Death and modern strains indicates that genetic factors are not causal for the catastrophic effects of the black death when compared to the less severe effects of modern plague [27]. Other influences, such as environmental, immunological and social factors, for example sanitation, could affect the severity of the disease. Wagner *et al.* [40] point out that sustained heavy rainfall predated all three plague pandemics, with the two historic pandemics concluding in climatically steadier periods, whilst the third is still ongoing. To trace the epidemiological history of the black death, Spyrou *et al.* [91] have sequenced more *Y. pestis* aDNA from early victims from Spain (1300–1420 CE). High similarity compared to the English paleogenome, combined with historical reports, support the theory of a single entry of the plague in the spring of 1348 near Barcelona coming from the lower Volga region of Russia through the Crimean peninsula [92]. A subsequent study by Spyrou *et al.* [4] supported the proposed single entry by reconstructing 34 s pandemic paleogenomes from Spain, Russia and Germany and finding a Russian sample that is ancestral to all second pandemic strains.

The subsequent 400 year duration of the second pandemic within Europe gives room for two possible explanations: long-term persistence of *Y. pestis* cycling within and between rodent and human populations, or repeated introductions [93]. With the first genomes stemming from the beginning of the second pandemic, Bos *et al.* [93] set out to answer that question by retrieving genomes from the late second pandemic. Victims dating to the 18th century in France were used to generate a *Y. pestis* paleogenome. A phylogeny constructed from available extant and ancient genomes groups the late second pandemic genome within a single lineage with no extant members. The most ancestral sequence in the lineage was recovered from a victim of the Black Death. Bos *et al.* therefore concluded that *Y. pestis* persisted in Europe rather than being continuously reintroduced. Spyrou *et al.* [4] later confirmed this notion by comparing 34 paleogenomes of the second pandemic, determining that all post-Black Death genomes stem from a single ancestral strain, supporting single entry. At least one plague lineage persisted in Europe, causing two major epidemics in 16th-century Germany and 18th-century France [4]. From Europe, *Y. pestis* travelled into Russia and eventually to China, the origin of the third pandemic [91].

The third pandemic started in the mid-19th century and disseminated worldwide through marine routes, with active foci remaining today in Africa, Asia and the Americas [94]. Cui *et al.* [95] collected 133 genomes of the modern pandemic from Asia and included 2 aDNA samples (from Haensch *et al.* [96] and Bos *et al.* [27]) to look at mutation rate variation in *Y. pestis *. Rate estimates varied 40-fold between lineages, despite recent emergence and slow mean evolutionary rates [95]. Harbeck *et al.* [97] also reported large rate variation, as they found no temporal structure when regressing genetic distance against sample date in a phylogeny of modern and ancient strains. The variation is attributed to changing replication rates, fluctuating selection pressures, or a combination of the two. When looking at the phylogeny of *Y. pestis *, there are a high number of polytomies, which Cui *et al.* ascribed to bursts of fixed mutations in narrow time windows. Feldman *et al.* [86] report that paleogenomes from the Justinian plague are further from the root of the phylogeny than samples from the Black Death or modern period despite being older. The increased variation in the Justinian lineage is attributed to a rate acceleration and geographical expansion during epidemic spread [4]. Feldman and colleagues ensure that the reported rate is based on accurate single-nucleotide polymorphism (SNP) calling by using rigorous criteria such as at least five times coverage and presence in at least 90 % of sequences obtained. When applying the same criteria to the paleogenome previously generated by Wagner *et al.* [40], 66 out of 176 SNPs were not called, highlighting the difficulty of generating accurate rate estimates from low-coverage paleogenomes. Increased substitution rates during epidemic spread were also reported for the second pandemic by Spyrou *et al.* [4]. Large underlying rate variation has led to differing estimates for genome-wide substitution rates and therefore the dates of the emergence of pathogenic *Y. pestis* [98]. The most recent common ancestor of all extant *Y. pestis* was estimated to be 1505–6509 YBP by Cui *et al.* [95] in a study using contemporary DNA. The estimate was revised to 5021–7022 YBP by Rasmussen *et al.* [85] with four paleogenomes, the oldest stemming from 5000 YBP. The most current estimate was calculated by Spyrou *et al.* [89] at 5299–8743 YBP using 20 paleogenomes dating back to 3800 YBP. Due to the time-dependent rate phenomenon, dating based on extrapolation from contemporary sequences alone is problematic [33]. Therefore Cui *et al.’s* estimate is likely to be less robust than Spryou *et al.*’s estimate. Recurring updates in date estimates highlight the difficulty of establishing a definitive timeline for bacterial evolution, especially without using aDNA. When using aDNA, however, the paleogenome used for calibration must be of sufficient coverage to avoid false-positive SNPs [86].

**Fig. 3.**
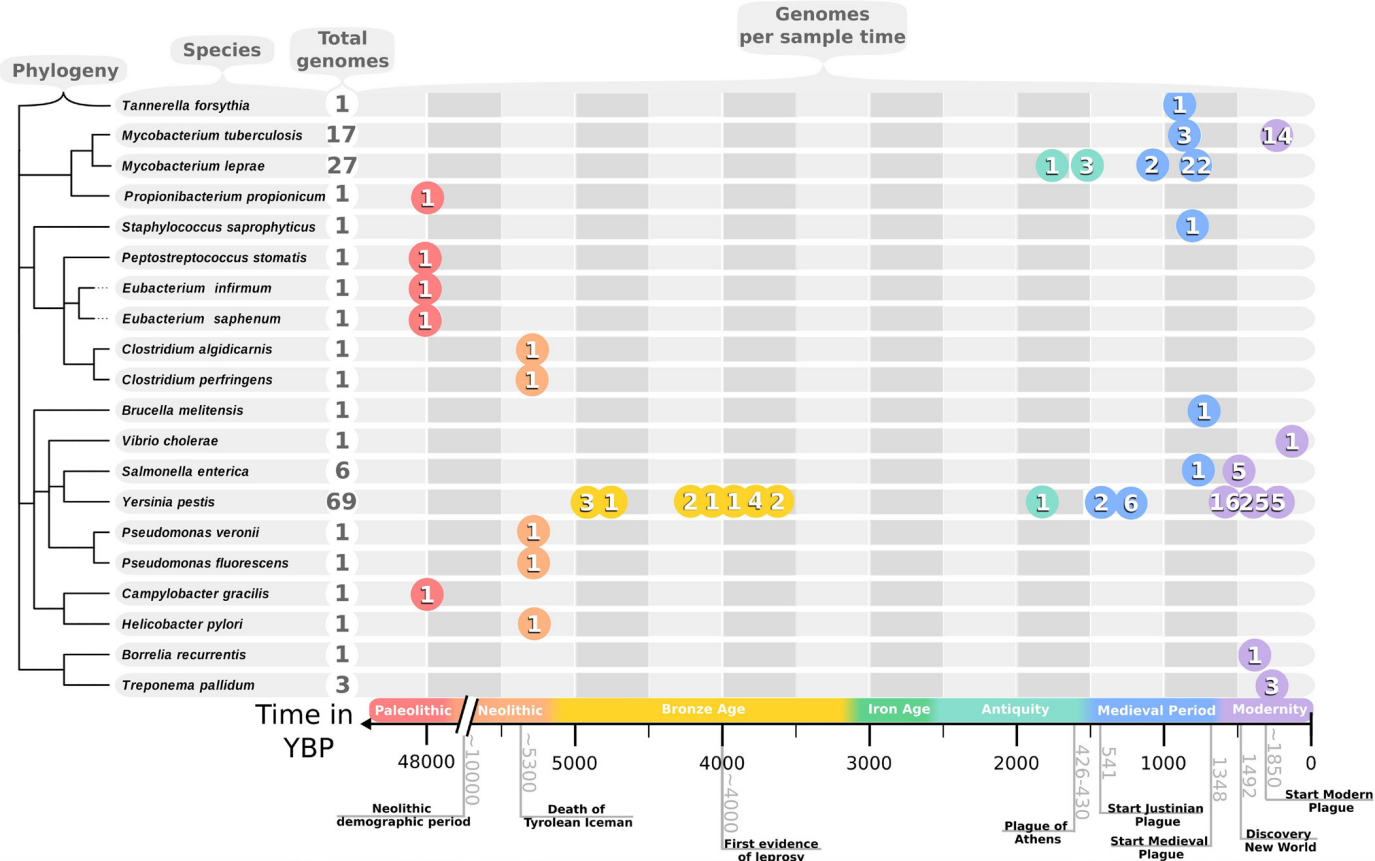
Catalogue of available bacterial paleogenomes:. The phylogenic relationship between the species from which the paleogenomes were reconstructed as inferred using the NCBI Taxonomy Common Tree tool is displayed on the left of the figure. The total quantity of available genomes is listed after the species name. On the right the number of genomes per species and historical time period is displayed, with significant events highlighted on the timeline at the bottom.

*Y. pestis* is a slowly evolving and well-studied pathogen with a well-documented historical record and an abundance of paleogenomes available (69 as of 2019; see Fig. 3). However, it still lacks a reliable evolutionary timeline, highlighting the difficulty of charting the bacterial past. Central for the analysis of *Y. pestis* with aDNA and the entire paleogenetic field was the 2011 study by Bos and colleagues [27], which provided the first bacterial paleogenome from four English plague victims from a single cemetery. The 2019 study by Spyrou *et al.* [4] illustrates what remarkable progress has been made in only 8 years by providing 34 full paleogenomes from all over Europe.

#### *Mycobacterium tuberculosis*

*M. tuberculosis* is the principal causative agent for TB, which is widespread today, with 10 million active cases in 2016 and an annual mortality of 1.7 million [99]. Formerly, TB was even more common in Europe [100], with Robert Koch claiming that it accounted for one in seven deaths in 1882 [101]. *M. tuberculosis* has co-existed with its host over millennia, witnessed by skeletal evidence in Neolithic humans [102]. Bone lesions can form in three to five per cent of individuals and are marked by spinal lesions and destruction of vertebral bodies, potentially leading to fusion of vertebrae [102]. The diagnosis of tuberculosis from bone is complicated by other diseases manifesting similarly on the spine, such as brucellosis, fungal infections, pyogenic ostemyelitis and neoplastic growths [103]. Paleopathologists can suggest a likely diagnosis by combining the analysis of multiple skeletal lesions [103]. Generally, bone lesions are insufficient to diagnose tuberculosis with certainty, but are an indicator to aDNA researchers that bacterial genetic material could be present, thereby increasing the chance of successful extraction [103]. PCR amplification of bacterial aDNA was used to verify cases of *M. tuberculosis* infection from skeletal lesions in the submerged village of Atlit-Yam, one of the first settlements showing evidence of agriculture and animal domestication in the Mediterranean around 9000 YBP [102]. The characteristic deletion of TbD1 present in the Atlit-Yam samples indicated the presence of the modern lineage of *M. tuberculosis* at 9000 YBP. However, verification through PCR amplification from bone samples is ambiguous, since multiple species of the genus *Mycobacterium* are common in the environment and can colonize postmortem [10]. Without sequencing of the amplification product and querying a *Mycobacterium* database, the authenticity of the findings cannot be presumed [10], making the Atlit-Yam study questionable. Skeletal evidence backed by PCR amplification identified likely tuberculosis in 7000-year-old skeletons from Hungary [104]. Lesions were found in pre-dynastic Egyptian remains dated 5000 YBP [105] and in the earliest cattle breeders in Sweden dated 5000 YBP [106], and these were further analysed with PCR. In addition to studies of ancient humans showing signs of TB indicating a long-entangled history of *M. tuberculosis* and modern humanity, aDNA has also been PCR-amplified from an 18 000-year-old extinct bison from North America, but this finding awaits independent authentication [107].

Claims for the existence of TB in the Americas predating the colonization by Europeans based on skeletal lesions alone has long been controversial, as described above [46]. Analysis of modern strains of *M. tuberculosis* supports the similarity of American and European strains, implying post-contact emergence of TB in the Americas [108]. Salo *et al.* [46] successfully amplified *M. tuberculosis* from a 1000-year-old pre-contact Andean mummy to test the pre-contact hypothesis. Bos *et al.* [39] delivered unambiguous proof by reconstructing a paleogenome from Peruvian skeletons and implicated sea mammal carriers in its appearance in the Americas. The failure of pre-contact strains to group with extant American lineages indicates a replacement by post-contact strains, which also explains the modern similarity between American and European strains [39].

Research into *M. tuberculosis* was further propelled by making use of a sealed crypt that was discovered in Hungary in 1994, revealing numerous naturally mummified bodies dating to 1731–1838 and indicated to be victims of TB by contemporary archives [109]. Soft tissues and bones were uncovered along with archives describing symptoms during an endemic period of the disease. Initial PCR amplification suggested that 55 % of individuals were infected with TB [109]. Chan *et al.* [110] were the first to apply metagenomics to the human remains using lung tissue from a single mummy to generate an *M. tuberculosis* paleogenome that revealed mixed infections with two different strains similar to contemporary German strains. Kay *et al.* [100] carried out a more comprehensive study and were able to reconstruct 14 complete paleogenomes for *M. tuberculosis* by using HTS on 26 bodies. A phylogeny was constructed to understand the relationship between modern strains and *M. tuberculosis* of the 18th century, where TB was more lethal. All ancient samples grouped into a single lineage containing some contemporary strains. The most recent common ancestor of this lineage was dated to the late Roman period (396 CE) [100]. The membership of some contemporary strains among the ancient lineage rules out a scenario similar to the post-contact replacement of TB in the Americas [95]. Infections with multiple strains in the mummy were observed, which possibly contributed to higher historical mortality, as they are now rarely found [100].

To date the emergence of TB, Bos *et al.* [39] used aDNA and arrived at an estimate of less than 6000 YBP (rate=0.046 Mby^−1^), which was supported by Kay *et al.* [100], who report similar mutations rates (0.05 Mby^−1^) with their paleogenomic approach. This dating would place the recent bound of the emergence of tuberculosis after the Neolithic demographic transition, possibly emerging through zoonotic transfer from livestock. Within the paleogenetic efforts to uncover the past of TB, the Bos *et al.* [39] study is a key accomplishment, having not only proved the pre-contact existence of TB, but also finding a potential mode of travel from Europe to the Americas. The carriage of human disease from Europe to the Americas by sea mammals is an exceptional find and one of the most intriguing anecdotes told by ancient pathogen genomics. Amongst retrievals of bacterial paleogenomes, the study by Kay and colleagues [100] should be highlighted, as they were able to retrieve 14 paleogenomes without targeted sequencing, using a metagenomic HTS approach instead. The successful construction of paleogenomes from metagenomic sequencing is exceptionally useful for providing an unbiased view of ancient bacterial communities.

#### *Mycobacterium leprae*

Hansen [111] was the first to discover that *M. leprae* was the causative agent for leprosy [112]. Leprosy is a chronic granulomatous infection of the skin and peripheral nerves causing sensory and motor impairment accompanied by deformities and disabilities [113]. As one of the oldest recorded and most stigmatizing infections in history, it is of prime interest to paleomicrobiology [114].

The first historical records describe symptoms of leprosy in China, India and Egypt about 600 BC [115]. The oldest archaeological evidence, identifying leprosy-specific bone lesions in a 4000-year-old skeleton, points towards an origin in India [116], wherefrom leprosy is thought to have spread to Greece through returning troops of Alexander the Great [115]. Romans then brought it to the Mediterranean, and finally it dispersed to the rest of Europe from Italy [117]. In medieval Britain, leprosy was widespread until a gradual and unexplained decline starting in the 14th century and continuing to the 16th century [118]. To investigate the underlying reasons for the decline of leprosy, Schuenemann *et al.* [114] extracted aDNA from multiple medieval English samples. The study investigated whether a decrease in virulence might be explained by genetic changes, using 10 paleogenomes from a broad sample range (fifth−14th century). Previously, Schuenemann *et al.* [38] reconstructed paleogenomes of medieval leprosy cases from the UK, Sweden and Denmark with Mendum *et al.* [118], providing a further two paleogenomes from the same sample. All three studies generated paleogenomes that all have close modern-day relatives, suggesting that genetic factors are not causal for the decline [38, 114, 118]. Krause-Kyora and colleagues [119] recovered aDNA from Danish skeletons from the 13th–16th century to address the decline and looked at the correlation with major leprosy susceptibility factors in the human leucocyte antigen region. Comparisons of *M. leprae *-positive and -negative cases uncovered a significant association of the allele DRB1*15 : 01 in leprae victims, which is a known susceptibility factor in modern cases. The stigmatization and the forbidding of marriage for the infected, combined with a hormone-related decrease in fertility through leprosy, might have led to negative selection on the DRB1*15 : 01 allele [119]. Donoghue *et al.* [44] point at both *M. tuberculosis* and *M. leprae* being widespread Europe during the first millennium CE, with only leprosy declining drastically thereafter. To test whether this is due to cross-immunity, Donoghue *et al.* carried out PCR amplification targeting both species in archaeological samples from the Roman period to the 13th century. Forty per cent of the samples showed co-infection, possibly indicating that an increase in mortality from TB led to a tandem decrease in leprosy.

The dating of the most recent common ancestor of *M. leprae* appears to differ depending on how many aDNA samples are included. The most comprehensive study conducted by Schuenemann *et al.* [114] arrived at an estimate of 4515 YBP, with a mean evolutionary rate of 0.0075 Mby^−1^. This estimate is lower (rate=0.0086 Mby^−1^) than their previous findings incorporating fewer medieval and modern strains [38]. Benjak *et al.* [120] report a lower estimate (rate=0.0078 Mby^−1^) using five medieval strains intermediate between the two studies by Schuenemann *et al.* The more medieval strains that are included, the older the estimate for the most recent common ancestor, suggestive of the time-dependent rate phenomenon.

#### *Salmonella enterica*

*S. enterica* serovar Typhi causes typhoid fever, which is a considerable public health problem for developing countries and causes 17 million new infections and 600 000 deaths each year [121]. Interest in typhoid fever from a paleomicrobial perspective was sparked when the plague of Athens was linked to *S. enterica* [122], which was historically attributed to a multitude of infections: Ebola, anthrax, plague, influenza, smallpox, tuberculosis, scarlet fever, Lassa fever, measles and typhoid fever [122]. Papagrigorakis *et al.* [122] targeted a range of aetiological agents of putative underlying diseases both bacterial and viral and extracted aDNA they deemed similar to *S. enterica* sv. Typhi. Shapiro and colleagues [123], however, upon reanalysis of the same sequence, found it to group outside both *S. enterica* and *S. typhimurium *, conceivably still a species of *Salmonella* but not typhoid. Shapiro *et al.* suggested that the sequence corresponds to a modern, free-living soil contaminant, whose presence has been overlooked due to lack of rigorous controls.

The first aDNA *S. enterica* was generated by Vågene *et al.* [124] from teeth found in a Mexican cemetery that was linked to the cocoliztli epidemic of 1545–1550 through historical documents. Cocoliztli is one of the principal epidemiological events of the drastic post-contact population decline in the 16th century Mesoamerica, which was of unknown origin. Through untargeted HTS and a comparison to an extensive pathogen database followed by targeted capture, Vågene *et al.* were able to link cocoliztli to *S. enterica* serovar Paratyphi C. Like Typhi, Paratyphi C causes enteric fever and is transmitted via the faecal–oral route [124]. It has been suggested that lifestyle alterations under Spanish subjugation, such as coerced relocations, changing domestic arrangements and novel farming practices, could have compromised hygiene and facilitated Paratyphi C transmission [124].

Zhou *et al.* [125] provided another genome for *S. enterica* sv. Paratyphi C from 800-year-old bones and teeth from a single Norwegian skeleton. Zhou *et al.* called the novel strain Ragna and calculated evolutionary rates for the Paratyphi C lineage, including the paleogenome, arriving at a median rate of 0.079 Mby^−1^. The most recent common ancestor of all Paratyphi C genomes was estimated to have existed 1162–1526 YBP, the split of Ragna from the other lineages occurring at 456–664 YBP. Seventy-eight per cent of genes were intact core genes with uninterrupted open reading frames and only 604 SNPs (rate=0.08 Mby^−1^) separated Ragna and the most recent common ancestor of modern Paratyphi C. Zhou *et al.* note that a scarcely changed genome even after 800 years of evolution is surprising, as local ecological interaction might plausibly cause variation in gene content.

Within bacterial paleogenetics the work of Vågene and colleagues [124] is a key publication, as it shows how uniquely positioned aDNA is to attempt to answer questions about the human past. The underlying cause of the cocoliztli epidemic remained unknown due to the lack of a written evidence, with only ambiguous depictions of symptoms available [124]. Paleogenetics thus proved invaluable in providing a possible aetiological agent where other disciplines had exhausted their means.

#### *Treponema pallidum*

*T. pallidum* can cause a wide range of diseases, of which syphilis and yaws cause the highest disease burden, with more than 300 000 new yaws infections being reported between 2008 and 2012 and syphilis resulting in 10.6 million cases worldwide in 2009 [126]. Treponematoses, the umbrella term for all diseases caused by *T. pallidum *, can cause skeletal marks like TB and leprosy, making it of great interest to paleopathologists [82]. As for TB, diagnosis based on skeletons alone is challenging. Lesions can easily be mistaken for postmortem bone damage, even by experienced anthropologists, which has led to the same skeletons being reported both as victims of treponematoses and free of the diseases in different reports [127]. Skeletal evidence and historical records suggest an American origin for syphilis, with worldwide transmission starting in the 15th century [126]. Some sources pinpoint the origin of European syphilis to Barcelona in the period immediately following the return of Columbus [128], leading historians to suggest the introduction to Europe through his returning crew [129, 130]. However, Rothschild *et al.* [131] found signs of trepanomal infection in a Pleistocene bear (11 500 YBP), pointing to an Old World origin. Harper *et al.* [132] questioned this finding, pointing out that the lesions are non-specific to treponematoses. With archaeological evidence alone, the origin of syphilis in the Old World remains contentious. Kolman *et al.* [133] were the first to successfully amplify *T. pallidum* aDNA using PCR from a 200-year-old specimen from Easter Island. The study remained the only successful attempt until 2012, was based on PCR amplification alone without subsequent sequencing, and therefore lacks authentication [134]. The acquisition of bacterial aDNA from *T. pallidum* subsequently proved difficult, as several failed paleomicrobial studies illustrate [135–137]. The aDNA drought was ended when Montiel *et al.* [134] analysed neonatal remains exhibiting skeletal lesions. The samples came from a crypt used in 15th and 16th century Spain from the same region the Columbus exhibitions departed. The location, dating and presence of skeletal lesions made the existence of *T. pallidum* highly likely. Schuenemann *et al.* [126] provided the third study with aDNA generating three paleogenomes of *T. pallidum* retrieved from a burial site used between 1681 and 1861. Out of five excavated individuals, only three contained aDNA from *T. pallidum.* A phylogeny of the recovered paleogenomes together with 39 extant genomes revealed that 2 grouped with syphilis strains, while the other grouped with a yaws-causing strains. Thus Schuenemann *et al.* [126] apparently recovered the first aDNA from a yaws infection.

As all paleomicrobial studies on *T. pallidum* are from post-contact samples, and because of the difficulty of obtaining aDNA, the origin of syphilis in Europe has not yet been resolved. However, Montiel and colleagues’ work [134] should be noted for laying the groundwork in sample choice for potential future studies to resolve this outstanding question just outside the grasp of current paleogenetic efforts.

#### *Vibrio cholerae*

*V. cholerae* is the aetiological agent of cholera, with a total of seven pandemics documented since 1817 [138]. The first pandemic originated on the Indian subcontinent, while the seventh started in the early 1960s in Indonesia and is still circulating today [83, 139]. In 2012, *V. cholerae* infected 3–4 million people and killed nearly 100 000, showing that *V. cholerae* still has a high disease burden [50]. The drivers of the modern outbreak remain elusive, with the predominant pathogenic strain harbouring two genetically distinct biotypes: classical and El Tor [50]. Stored *V. cholerae* strains are available for the sixth (1899–1923) and seventh pandemics. The stored strains show a replacement of the classical biotype by El Tor prior to the present pandemic [140]. Devault *et al.* [50] were able to reconstruct a *V. cholerae* genome from a preserved intestine. The intestine belonged to a second pandemic 1849 cholera outbreak victim, showing sequence similarity to extant genomes of the classical biotype, indicating a common origin.

The emergence of the El Tor strain was dated to between 1940 and 1957, with the classical strain putatively emerging between 1843 and 1860 [50]. A strict clock of 0.083 Mby^−1^ was used for dating, which was estimated earlier by Mutreja *et al.* [141], who regressed root-to-tip distance against isolation date of 154 contemporary genomes. The origin of pathogenic *V. cholera* was estimated to be 430–440 YBP by Mutreja *et al.*, who highlighted that recombination and mutational saturation biases clock rates and thus date estimates.

#### *Helicobacter pylori*

*H. pylori* is found in the stomach of nearly half of the human population [142] and whilst most are asymptomatic carriers, approximately 15 % develop peptic ulcers and 0.5–2 % acquire gastric adenocarcinoma [143]. Longstanding coevolution enables the tracing of ancient and modern human migration through the *H. pylori* phylogeny [144]. Linz *et al.* [145] showed that genomic divergence is negatively correlated with geographical distance from East Africa, indicating a shared out-of-Africa origin with humans 58 000 YBP. The first indirect aDNA assessment of the bacterium was made when Allison *et al.* [146] amplified antigens of *H. pylori* from the faeces of Andean mummies dated to 3000 YBP. Castillo-Rojas *et al.* [147] were able to confirm pre-contact existence by amplifying part of the 16S rDNA gene from a Mexican mummy dated to 1350. Whereas the study by Allison and colleagues lacked sequencing and therefore is unauthenticated, Castillo-Rojas *et al.* performed sequencing and alignment, lending their claims more weight. Swanston *et al.* [143] amplified virulence-associated *H. pylori* genes from the stomach of 200–300 year-old individuals recovered from a glacier in Canada. Swanston *et al.* found genetic similarities to European isolates, indicating European strains were present in First Nation territory 200–300 YBP. The first paleogenome of *H. pylori* was retrieved from the 5300-year-old Tyrolean iceman by Maixner *et al.* [148]. The paleogenome exhibits close similarity with the Asian population, indicating that the European strains are of Asian origin subject to African admixture within the past few thousand years [148].

#### Other pathogens

*Brucella melitensis* causes brucellosis infecting livestock, from which it transmits to humans, generating 500 000 new cases annually [149]. In humans it can cause vertebral lesions, which, unlike those of TB and trepanomatoses, are specific to the disease [150]. Potential *B. melitensis *-associated skeletal marks were identified in a skeleton of *Australopithecus africanus* dated to 2.4 to 2.8 million YBP, suggesting a long association with hominids [151]. Kay *et al.* [149] generated a paleogenome of *B. melitensis* from a 14th-century Italian skeleton through untargeted HTS. The genome groups most closely with extant Italian strains, showing a continuity of infection in Italy.

Devault *et al.* [152] conducted an untargeted HTS approach on calcified abscesses on a skeleton from a Late Byzantine-era cemetery in the ancient city of Troy. The metagenome of the skeleton, dated to 1154–1224 CE, was dominated by *Staphylococcus saprophyticus *, a bacterium typically causing urinary tract infections. The bacterium *Gardnerella vaginalis* was also found, which commonly occurs in modern-day pregnancy-related infections.

*Borellia recurrentis* is carried by the human body louse *Pediculus humanus* and causes louse-borne relapsing fever, which has disappeared in the Western world but formerly caused millions of deaths in Europe [153]. Guellil *et al.* [153] were able to retrieve a paleogenome from a Norwegian skeleton dated to 1430–1465.

The recovery of the paleogenomes of *S. enterica* and *B. recurrentis* are major achievements, as neither leave visible marks of skeletons or mummies, compounding the challenges for ancient pathogen research by making a targeted approach difficult [153]. However, Devault and colleagues [152], like Kay *et al.* [149], showed that an untargeted metagenomic approach can lead to the reconstruction of a high-coverage paleogenome.

### Human microbiome

Paleomicrobiology has not stopped at the recovery of individual genomes, but has also assessed ancient microbiomes. Humans carry microbes comprising thousands of different species that collectively outnumber our own cells by at least an order of magnitude [154, 155]. The longstanding interaction of these co-inhabitants of our bodies has led to a growing appreciation of the human microbiome in studying human evolution. The focus of research has shifted away from individual pathogenic microbes towards an investigation of microbiomes as mutualistic components of metabolism, immune development and neurological function [155]. Ancient microbiome studies have drawn primarily from three sources– mummified remains, coprolites and dental calculus – and can be separated into studies focusing on oral or gut microbiomes [156].

The evolution of the oral human microbiome can be assessed through dental calculus and is primarily shaped by two major shifts in human diet: a shift towards carbohydrate-rich diets in the Neolithic demographic transition (~10 000 YBP) and the arrival of industrially processed flour and sugar (ca. 1850) [72]. Adler *et al.* [72] used HTS on dental calculus from 34 teeth dating from before the development of agriculture to the mediaeval period, showing that abandoning the hunter–gatherer lifestyle led to a disease-associated reconfiguration of the mouth microbiome. Warinner *et al.* [67] analysed dental calculus from the teeth of four German individuals from 950 to 1200 CE showing mild to severe periodontal disease. The sample was dominated by the so-called red complex pathogens, a group comprising *Tannerella forsythia *, *Porphyromonas gingivalis* and *Treponema denticola *, which supply the most important pathogens in adult periodontal disease [157]. Besides long-term carriage of opportunistic pathogens, Warinner *et al.* reported the presence of genes homologous to putative antimicrobial resistance genes. Weyrich *et al.* [158] analysed dental calculus of five Neanderthal specimens, a historic wild-caught chimpanzee and a modern human. The oral microbiome of the Neanderthal was closer in composition to that of the historical chimpanzee than the microbiome of modern humans. Pathogens associated with caries, causing the decay of teeth, such as *S. mutans* and members of the red complex were found in the Neanderthal samples [158]. The paleogenomes o*f Campylobacter gracilis*, *Propionibacterium propionicum *, *Fretibacterium fastidiosum *, *Eubacterium infirmum *, *Peptostreptococcus stomatis* and *Eubacterium sphenum* were retrieved from the Neanderthal extractions.

The evolution of the gut microbiome can be assessed from both coprolites and mummified organs, which was successful early in paleomicrobiology. Ubaldi *et al.* [45] paved the way by proving that 16S rDNA of multiple species can be amplified from 900 to 1000 year-old Andean mummies. Ubaldi *et al.* predominantly discovered bacteria of the genus *Clostridium *. Cano *et al.* [47] amplified 16S rDNA of a broad range of bacteria from the 5300-year-old Tyrolean iceman mummy. The stomach was entirely composed of *Burkholderia picketii*, an aquatic bacterium possibly originating from meltwater, entering the body. The colon harboured several *Clostridium* species characteristic of the contemporary human faecal flora. Rollo *et al.* [73] conducted a study analysing the persistence of bacterial DNA, comparing the Tyrolean iceman to a mummy dated to 1918. Rollo and colleagues showed that the characteristic composition of extant microbiota is still present in the younger mummy. However, even under almost ideal conditions of preservation, the 5300 year old sample is almost entirely composed of *Clostridium. Clostridium* species are likely to persist better as they are endospore-forming, low-GC, Gram-positive bacteria, which are all qualities previously described as beneficial for preservation by Willerslev *et al.* [57]. Anaerobic species like *Clostridium* also survive due to a rapid decrease in oxygen concentration postmortem, indicating that the uncovered abundance of *Clostrdium* is unlikely to reflect the true state of the microbiome at the point of death [73]. Luciani *et al.* [159], upon analysis of the same Andean mummy as Ubaldi *et al.*, found the pathogen *Haemophylus parainfluenzae*. Santiago-Rodriguez *et al.* [160] contributed the third study on the Andean mummy using HTS to characterize the microbiome, finding putative antimicrobial resistance genes. They also found an abundance of *Clostridium *, which comprised up to 96.2 % of the species in the gut. Lugli *et al.* [161] reanalysed the Tyrolean iceman using HTS data generated by Maixner *et al.* [148] and predominantly found *Clostridium* and *Pseudomonas *. Lugli *et al.* were also able to reconstruct five paleogenomes of the specie*s Clostridium perfringens, Clostridium algidicarnis*, *Pseudomonas veronii* and *Pseudomonas fluorescens* and a genome of the genus *Clostridium* that could not be assigned a species. The frequent retrieval of clostridial species could be attributed to their better preservation. However, many members of the genus are also found in soil [162]. Retrieval of clostridia from ancient samples could be false positives resulting from sequence similarity with soil-dwelling relatives that colonize postmortem and subsequently acquire PMD sequence patterns. Although bacteria colonizing after death can be genuinely ancient, they are misleading concerning past microbiota and the humans that harboured them. The continual reuse of mummified samples in aDNA gut microbiome research shows how difficult the acquisition of viable specimens is. Fortunately for paleomicrobiology, coprolites are another source for gut microbiomes.

Tito *et al.* [63] first analysed two coprolite samples dated to 1300 YBP using HTS and found a dominance of the phyla *Bacteroidetes *, *Firmicutes *, *Actinobacteria* and *Proteobacteria* typical of contemporary faecal microbiomes. The two ancient samples were more genetically alike than modern samples, possibly owing to biogeographical structuring. Tito *et al.* suggest that microbiomes were formerly more geographically structured, possibly due to more locally restricted diets. Cano *et al.* [59], through the analysis of coprolites dated to 180–600 CE, showed that the extinct Amazonian Huecoid and Saladoid cultures could be distinguished based on their coprolites. The difference possibly stems from host genetics or differences in diet [59]. Tito *et al.* [66] conducted the most extensive study to date, incorporating coprolites from three archaeological sites ranging from 8000 to 1400 YBP. A source analysis showed that two of the sites were either of unknown source or more alike to compost than human gut. The remaining Mexican sample, which featured in their previous study [63], showed higher similarity to contemporary rural microbiomes than urban communities. A modern lifestyle may have influenced ancestral mutualistic ties between humans and bacteria, possibly increasing the risk of autoimmune diseases amongst other conditions [63].

The study of Weyrich and colleagues [158] shows how rich of a resource calculus is, having constructed paleogenomes from six different species. The importance of the study also lies in the age of the paleogenomes, which date to 48 000 YBP, and are thus by far the oldest bacterial genomes, with the next most ancient paleogenomes dating to 5300 YBP [161]. The temporal reach of dental calculus is only surpassed by ice cores, which are the only source of environmental bacteria.

### Non-human associated bacteria

Most environmental aDNA comes from permafrost due to the excellent conditions for preservation. Whether aDNA yielded from ice can be compared with other aDNA samples is questionable, as microbes could potentially remain metabolically active within micro-encasings of liquid water [74]. Active bacteria could potentially repair their DNA, influencing PMD signatures [74]. Frozen samples of potentially active bacteria could be a reservoir of adaptive potential upon thawing of the ice, given the microbial propensity to transfer genetic material horizontally. Bidle and colleagues [74] point out that the pace of evolution after major global glaciations seems to be increased. The first environmental samples of bacterial aDNA were provided by Priscu *et al.* [53] from an ice layer dated to 1 million YBP from Lake Vostok. Christner *et al.* [54] amplified 16S rDNA in five 20 000-year-old ice cores and found most bacteria to be related to *Bacillus* and *Actinomycetes *. Willerslev *et al.* [57] used 16S rDNA amplification on ice dated up to 800 000 YBP and found it dominated by *Actinobacteria *. Bidle and colleagues [74] used 16S rDNA amplification on Antarctic ice belonging to the oldest known samples reaching up to approximately 8 million YBP. The ice harboured *Alphaproteobacteria *, *Acidobacteria *, *Firmicutes* and *Cytophaga–Flavobacterium–Bacteroides* divisions. D’Costa *et al.* [163] investigated 30 000-year-old Beringian permafrost to look at genetic content through HTS. Genes coding for the resistance to beta-lactam, tetracycline and glycopeptide antibiotics were found, revealing ancient evidence of antimicrobial resistance. Segawa *et al.* [164] collected ice core samples from the Kyrgyz Republic dated to 12 500 YBP to look at the mutation rates of cyanobacteria through comparison to modern samples. Looking at the internal transcribed spacer region of two cyanobacterial operational taxonomic units, Segawa *et al.* found rates of 10 Mby^−1^.

Bidle and colleagues’ work [74] is exceptional within paleogenetics, as it provided the oldest genetic evidence of bacteria at 8 million YBP. The study of environmental aDNA has thus spanned larger time periods than was ever possible by analysing human remains, although no bacterial paleogenome has been reconstructed from environmental aDNA to date, underlining the inherent challenges at this frontier of ancient DNA research.

## The future: avenues of research in bacterial aDNA

The future of bacterial aDNA research will continue to be punctuated by spectacular findings and technological advances that are difficult to foresee, such is the nature of discovery. Yet putting aside predictable trends for improved sample sizes, chronological coverage, aDNA recovery techniques, authentication methods and sequencing technology – important though they are – it is possible to chart out more broadly the areas where bacterial aDNA research stands to make great inroads into the study of bacterial evolution.

The unique contribution of aDNA to the study of evolution is the ability to replace inference about the past with evidence. The repertoire of available paleogenomes (Fig. 2, Table 1) facilitates more comprehensive studies, but is limited in its number of species and patchy in temporal resolution, with unexplored time periods such as the Iron Age (~3000–2500 YBP) and the Neolithic demographic transition (~10 000 YBP). However, analyses of modern genomes alone are uncertain and sometimes the most parsimonious inferences are erroneous. For instance, the notion that tuberculosis was first brought to South America by European colonialists, supported by the similarity of modern European and South American strains, was overturned by the discovery of archaic *M. tuberculosis* in a 1000 year old Andean mummy [46]. The future of aDNA is the systematic incorporation of densely sampled ancient genomes into analyses of modern genomes to produce a richer, and more accurate, understanding of microbial evolution.

**Table 1.** Table of available bacterial paleogenomes. The table shows all publications generating bacterial paleogenomes of >1.0-fold coverage. The respective sources that were used in the studies and the species from which the paleogenome was extracted are also listed

Species	First author and reference	Year published	Source	No. of paleogenomes
*Borellia recurrentis*	Guellil [153]	2018	Bones	1
* Brucella melitensis *	Kay [149]	2014	Bones	1
* Clostridium algidicarnis *	Lugli [161]	2016	Mummy	2
* Eubacterium infirmum *	Weyrich [158]	2017	Teeth	1
*Eubacterium sphenum*			Teeth	1
* Fretibacterium fastidiosum *			Teeth	1
* Campylobacter gracilis *			Teeth	1
* Peptostreptococcus stomatis *			Teeth	1
* Helicobacter pylori *	Maixner [148]	2016	Mummy	1
* Mycobacterium leprae *	Schuenemann [38]	2013	Bones	5
	Mendum [118]	2014	Bones	2
	Krause-Kyora [119]	2018	Bones	10
	Schuenemann [114]	2018	Bones	10
*** Mycobacterium tuberculosis ***	Bos [39]	2014	Bones	3
	Kay [100]	2015	Mummy	14
*** Peptostreptococcus stomatis ***	Weyrich [158]	2017	Teeth	2
*** Pseudomonas fluorescens ***	Lugli [161]	2016	Mummy	1
*** Pseudomonas veronii ***			Mummy	1
*** Salmonella enterica ***	Vågene [124]	2018	Teeth	5
	Zhou [125]	2018	Teeth	1
*** Staphylococcus saprophyticus ***	Devault [152]	2017	Bones	1
*** Tannerella forsythia ***	Warinner [67]	2014	Teeth	1
*** Treponema pallidum ***	Schuenemann [126]	2018	Bones	3
***Vibrio Cholarae***	Devault [50]	2014	Calcified pleura	1
*** Yersinia pestis ***	Bos [27]	2011	Teeth	1
	Wagner [40]	2014	Teeth	1
	Rasmussen [85]	2015	Teeth	2
	Feldman [85]	2016	Teeth	1
	Bos [93]	2016	Teeth	5
	Spyrou [91]	2016	Teeth	3
	Valtuena [87]	2017	Teeth	6
	Damgaard [88]	2018	Teeth	1
	Namouchi [168]	2018	Teeth	5
	Spyrou [89]	2018	Teeth	2
	Rascovan [169]	2019	Teeth	1
	Spyrou [4]	2019	Teeth	34
	Keller [90]	2019	Teeth	8

Broad questions continue to elude our understanding in bacterial evolution. What drives the structuring of pathogens into distinct strains or ‘clonal complexes’? Intense sequencing of decades-old freezer collections has revealed glimpses into an evolutionary dynamism in which apparently dominant contemporary strains are revealed to be transitory [165]. As in the case of tuberculosis in the Americas [46], the make-up of contemporary populations is an unreliable guide to the past, indicating that aDNA will be indispensable for understanding what forges bacterial population structure.

Understanding the historical context of bacterial evolution, particularly the evolution of new epidemic strains, is familiar territory for population genomics, yet densely sampled paleogenomes are needed to resolve persistent problems with molecular clock rates. In common practice, clock rates estimated over timeframes of years to decades are employed to infer deeper history by extrapolation, which is a risky occupation. Yet the time-dependent rate phenomenon renders this practice potentially fatal to attempts to accurately place bacterial evolution into the correct historical context. Consequently, we remain ignorant about the dates of deep splits in the phylogenies we routinely publish, and unable to draw safe inferences based on such dates. Establishing robust timeframes is crucial to determine, for example, whether AMR spreads due to rapid mobility, as described by Sheppard *et al.* [166], or whether AMR is ancient, as D’Costa and colleagues [163] suggest.

What are the genes and genetic variants that predispose bacteria to cause disease? Ancient genomes offer unique opportunities to understand the substitutions, and the sequence of evolutionary events that preceded major outbreaks. This is particularly valuable for mutations that were so successful that they subsequently became ubiquitous. Yet it is unlikely that bacterial paleogenomes will be incorporated systematically into genome-wide association studies because of concerns over (i) confounding factors between ancient and modern genomes, hosts and environments, and (ii) obtaining high-quality phenotypes. Instead, identification of potential virulence-conferring mutations is most likely to provide candidate loci directly for experimental studies.

More generally, how have human–microbe interactions influenced the evolution of each species? Understanding the landscape of the human microbiome prior to major transitions such as the Neolithic revolution and antibiotic revolution will be fundamental to progress. Recent discoveries in human and bacterial aDNA have highlighted this as particularly promising ground. aDNA studies have precipitated a major revision to our understanding of recent human and bacterial evolution, for example the spread of steppe ancestry across Eurasia, and the plagues carried with it [86]. The industrialization of aDNA research has begun in earnest in the human field, with over 2000 densely sampled human paleogenomes already sequenced [167], providing unique insights that in many cases have overturned previous evolutionary understanding. This is the next step for bacterial aDNA studies, a step that will permit us a gaze not only into our own past, but a glimpse into the inscrutable past of the oldest domain of life.
